# Evaluation of Eligibility Criteria Relevance for the Purpose of IT-Supported Trial Recruitment: Descriptive Quantitative Analysis

**DOI:** 10.2196/49347

**Published:** 2024-01-31

**Authors:** Romina Blasini, Cosima Strantz, Christian Gulden, Sven Helfer, Jakub Lidke, Hans-Ulrich Prokosch, Keywan Sohrabi, Henning Schneider

**Affiliations:** 1 Institute of Medical Informatics Justus Liebig University Giessen Germany; 2 Department of Medical Informatics, Biometrics and Epidemiology Friedrich-Alexander Universität Erlangen-Nürnberg Erlangen Germany; 3 Department of Pediatrics Medical Faculty and University Hospital Carl Gustav Carus TUD Dresden University of Technology Dresden Germany; 4 Data Integration Center Medical Faculty Philipps University of Marburg Marburg Germany; 5 Faculty of Health Sciences Technische Hochschule Mittelhessen University of Applied Sciences Giessen Germany

**Keywords:** CTRSS, clinical trial recruitment support system, PRS, patient recruitment system, clinical trials, classifications, data groups, data elements, data classification, criteria, relevance, automated clinical trials, participants, clinical trial

## Abstract

**Background:**

Clinical trials (CTs) are crucial for medical research; however, they frequently fall short of the requisite number of participants who meet all eligibility criteria (EC). A clinical trial recruitment support system (CTRSS) is developed to help identify potential participants by performing a search on a specific data pool. The accuracy of the search results is directly related to the quality of the data used for comparison. Data accessibility can present challenges, making it crucial to identify the necessary data for a CTRSS to query. Prior research has examined the data elements frequently used in CT EC but has not evaluated which criteria are actually used to search for participants. Although all EC must be met to enroll a person in a CT, not all criteria have the same importance when searching for potential participants in an existing data pool, such as an electronic health record, because some of the criteria are only relevant at the time of enrollment.

**Objective:**

In this study, we investigated which groups of data elements are relevant in practice for finding suitable participants and whether there are typical elements that are not relevant and can therefore be omitted.

**Methods:**

We asked trial experts and CTRSS developers to first categorize the EC of their CTs according to data element groups and then to classify them into 1 of 3 categories: necessary, complementary, and irrelevant. In addition, the experts assessed whether a criterion was documented (on paper or digitally) or whether it was information known only to the treating physicians or patients.

**Results:**

We reviewed 82 CTs with 1132 unique EC. Of these 1132 EC, 350 (30.9%) were considered necessary, 224 (19.8%) complementary, and 341 (30.1%) total irrelevant. To identify the most relevant data elements, we introduced the data element relevance index (DERI). This describes the percentage of studies in which the corresponding data element occurs and is also classified as necessary or supplementary. We found that the query of “diagnosis” was relevant for finding participants in 79 (96.3%) of the CTs. This group was followed by “date of birth/age” with a DERI of 85.4% (n=70) and “procedure” with a DERI of 35.4% (n=29).

**Conclusions:**

The distribution of data element groups in CTs has been heterogeneously described in previous works. Therefore, we recommend identifying the percentage of CTs in which data element groups can be found as a more reliable way to determine the relevance of EC. Only necessary and complementary criteria should be included in this DERI.

## Introduction

### Background

Clinical trials (CTs) are key to medical progress as they are used to implement a new therapy, a medical device, a diagnostic procedure, or a preventive measure [1,2]. CTs thus form an essential component of “translation,” the transfer of findings from basic medical research to clinical application [3].

An important part of planning a CT is to define criteria that all participants must meet. The main goal of these inclusion—and exclusion—or eligibility criteria (EC) is to specify the CT’s target population (ie, patients who have specific conditions and might benefit from the studied therapy). EC are also used to minimize disruptive factors that are under suspicion to interfere with the CT objectives. Additionally, EC consider individuals for whom a CT could pose a health risk, such as pregnant women. Finally, some EC are necessary for legal or organizational reasons [4,5].

The successful implementation of CTs depends on the recruitment of a suitable number of participants who fulfill all EC. Insufficient participant recruitment is the foremost reason for the premature discontinuation of CTs, which raises ethical concerns because participants are exposed to risk, without potential benefits. Furthermore, the extension of the recruitment period is also associated with significant financial costs and is consequently inefficient [6-10].

### Clinical Trial Recruitment Support Systems

Identifying individuals who fulfill all the EC of a study and are willing to enroll is challenging and time-consuming. In many cases, trial personnel manually search for suitable candidates in electronic health records (EHRs) [9,11]. A clinical trial recruitment support system (CTRSS), also called a patient recruitment system (PRS), can assist in increasing participant numbers [12-14]. These systems simplify study participant identification with secondary use of data already collected for care and billing purposes in clinics [15] and work by comparing EHR data with the specified EC of CTs. Most CTRSSs described in the recent literature are implemented for only 1 specific trial, medical department, or clinic, but there are also some approaches to develop a CTRSS that can be used for a wide range of CTs [14,16-22].

Several key considerations are necessary for the successful deployment of a CTRSS. First, to implement a CTRSS, it is necessary to have patient records as well as EC in machine-readable format to perform a comparison of both and create a list of potential participants [23,24]. The formatting of EC in machine-readable form depends on the underlying technology used. For instance, in a database-oriented system, the criteria can be developed using Structured Query Language (SQL). Previous research efforts have used ATLAS software (Observational Health Data Sciences and Informatics [OHDSI]) for this purpose. Artificial intelligence (AI)–based methods exist for translating ethical considerations from study protocols into ATLAS software, which can partially automate the process [25].

As previously stated, medical data from hospitals or medical centers are used to compare a CTRSS with the EC and shortlist individuals from the data pool who meet the EC for the study [26]. Consequently, a CTRSS can only specify search criteria that correspond to the existing data pool. Hence, to ensure efficient prefiltering of the data pool, the CTRSS must contain the maximum number of desired search criteria.

The consolidation of medical data in a centralized format remains a significant challenge in many systems due to limited data availability [27]. Additionally, many hospitals lack a retrievable centralized system for all accumulating medical data. Despite ongoing efforts to harmonize data, these approaches are not yet widely accessible. Therefore, establishing a connection to a comprehensive data repository that facilitates all the necessary search criteria is crucial in implementing a CTRSS, and a predetermined list of search criteria is essential.

### Eligibility Criteria

To characterize the necessary data for CTRSS implementation, multiple studies have examined the prevalence of data element groups in the EC of CTs. Even though the results of these examinations are heterogeneous, it is evident that diagnosis is the most frequent data element used in official study protocols, followed by data about therapies, medications, and diagnostic results [25,28-30].

When searching for potential study participants, there is often a need to manually search through a large number of patient files. Study personnel typically start by making a preselection. Initially, they check the EC that they consider most important, as this helps narrow down the pool of potential CT participants effectively. Some EC can only be assessed right before including participants in the study or necessitate a personal evaluation by the trial staff. One such criterion is the consent form that participants are required to sign during the inclusion process. Not all the EC specified in the study protocol are likely used for preselection in the context of a CTRSS [11]. When implementing a CTRSS, it is sufficient that only relevant data element groups be queried to obtain appropriate suggestions [30].

### Objectives

In previous work, we identified which data element groups are most commonly used in CT EC [30]. In this study, we investigated which of the data element groups identified in the previous studies, mentioned in the *Eligibility Criteria* section, are relevant in practice [25,28-30].

Another objective was to categorize these EC according to their underlying data element in order to identify the element groups, such as diagnosis, laboratory values, or demographics, that are most commonly used for patient recruitment, as well as those that are mostly irrelevant to a CTRSS. Since the use of different data elements and search algorithms is strongly influenced by the availability of this information in EHRs, we also investigated when a data element needs to be checked but is not available in the patient’s EHR.

The overall goal was to determine the relevance of data element groups for use in a CTRSS. Therefore, we wanted to find out how often different data element groups occur in CTs and how often the groups are considered relevant. We also wanted to determine how many studies these data element groups occur in.

## Methods

### Participants

Two groups of participants were enrolled in the study. The first group included 1 or more project participants (PPs) at each site who were responsible for data collection. These individuals had deep knowledge of medical informatics in general and were also involved in the development of a CTRSS.

The second group of participants were trial staff actively working in the field of study recruitment. They were referred to as trial professionals.

### Data Collection Sheet

To assess the relevance of data element groups, we first developed a data collection sheet to capture relevant information from the trial professionals. We wanted to capture all EC of the selected CTs in their original format, the underlying data element, and the assessment of study personnel in terms of relevance to patient recruitment. The development of the data collection sheet was an iterative process in which the categories were first discussed in a group of 10 CTRSS developers and then tested by 2 persons of this group on 3 randomly selected studies. In the next step, the group discussed any issues that arose. After 3 iterations, we achieved full agreement among all testers and developers.

For simple but unambiguous categorization, we used the 40 most common data element groups of EC from our previous work [30] and combined some rarely used groups into broader categories (eg, special laboratory information into a category laboratory value). If none of the given data element groups were appropriate, it was also possible to select a broader category, such as other procedural information, and provide a more specific description as a comment. These categories are then strongly linked to the data in EHRs and can therefore provide more insight into the possibilities of accessing elements of EC in clinical systems. For a complete list of all data element groups and other information composed on the data collection sheet, see Multimedia Appendix 1.

EC were classified into 3 CTRSS relevance categories: necessary, complementary, and irrelevant. Necessary items are those that determine the main selection of the desired cohort, complementary criteria in a manual process are mostly used in a second step to obtain more precise results, and irrelevant criteria are not used at all. Because there are criteria that may be important for participant selection but are not regularly documented in the EHRs and therefore must either be known by the treating physician or verified by direct questioning of the patient, we added the categories “necessary, not documented“ and “complementary, not documented.” In addition, there are various reasons criteria may be irrelevant. Therefore, we decided to add the categories “irrelevant, redundant” and “irrelevant, recorded at the time of enrollment.” The category descriptions are summarized in Table 1.

**Table 1:** Tabular view of EC^a^ relevance categories with descriptions.

Relevance category	Description
Necessary	Used for the main filter criterion, for example, the main diagnosis under investigation
Necessary, not documented	Used for the main filter criterion but cannot be checked on paper or digitally
Complementary	Used for the secondary filter criteria used to achieve more precise search results
Complementary, not documented	Used for the secondary filter criteria but cannot be checked on paper or digitally
Irrelevant	Not relevant for the participant search
Irrelevant, recorded at the time of enrollment	Not relevant, because it is only relevant after the initial participant search during the process of enrollment
Irrelevant, redundant	Used for criteria listed twice (duplicated), one time marked as redundant

^a^EC: eligibility criteria.

In addition, we had a field to note surrogate data element groups that could be used if a criterion was not documented in the EHR system. For example, if a particular condition is not documented in a timely manner and there is a lab result that is indicative of that condition, that lab result can potentially be used as an alternative data element.

Taking an example CT on diabetes with the inclusion criteria of a diagnosis of diabetes, an elevated laboratory value, and consent to participate in the CT, as well as the exclusion of drug abuse, this can be classified as shown in Table 2. In this example, the presence of diabetes is the main inclusion criterion, so it is classified as “necessary” and the laboratory value is classified as “supplementary,” since this criterion usually applies to all patients with diabetes. Alcohol abuse can be diagnosed in the medical history but is usually not documented in the patient’s record, and therefore, it is an additional filter criterion but cannot be verified by inspection of the file. Alternatively, it is possible to find notes about possible alcohol abuse in the records of the patient’s medical history.

**Table 2:** Sample representation of a completed data extraction sheet.

Original description	Content (simplified)	Data element	Relevance assessment	Surrogate parameter
Participants with a confirmed diagnosis of type 2 or type 1 diabetes	Type 1 or 2 diabetes	Diagnosis	Necessary	—^a^
Patients with controlled diabetes (HbA1c^b^<9%)	HbA1c<9%	Laboratory result	Complementary	—
Willing to take part in the trial	Consent	Informed consent	Irrelevant, recorded at the time of enrollment	—
History of drug abuse within 1 year prior to screening	Drug abuse	Diagnosis	Complementary, not documented	Other medical history

^a^Not applicable.

^b^HbA1c: glycated hemoglobin.

### Selection of Trials

We collected CT information from 8 university hospitals in Germany between December 2021 and March 2022 using the data collection sheet described in the *Data Collection Sheet* section.

The participating centers conducted the CT selection, including only CTs that recruited participants prospectively. This means that the CTs actively searched for individuals and asked them to participate. Animal, biomaterial, and case-control CTs were excluded. Psychiatric and oncological CTs were also excluded due to organizational reasons. There were no further restrictions on the selection of medical specialties.

Trial centers were contacted by the PPs to inquire about participation in the study. If a positive response was received, the CTs recommended by the trial personnel were incorporated into the analysis. The trial professionals were also asked to participate in this study to obtain a real-world view of the CT recruitment process. The PPs had either face-to-face or video meetings with the trial professionals to discuss the process of identifying potential participants and to categorize each individual criterion based on both parties’ experiences. If a positive response was received, the CTs recommended by the trial professionals were discussed and incorporated into the analysis.

### Data Validation and Analysis

**Figure 1:**
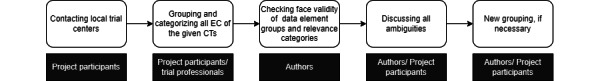
Sequence of data collection, with a mention of the groups of persons involved: contacting local CT centers (PPs), grouping and categorizing all EC of the given CTs (PPs and trial professionals), checking the face validity of data element groups and relevance categories (authors), discussing all ambiguities (authors and PPs), new grouping, if necessary (authors and PPs). CT: clinical trial; EC: eligibility criteria; PP: project participant.

All PPs were trained in a common training session where the data collection sheet was presented and tested. To ensure the correct use of the data element groups and the CTRSS relevance categories, we performed an additional step to validate the collected data: 2 authors went through all records and checked for consistency and face validity. If the entries were not fully understandable, they contacted the responsible PPs and discussed the case until agreement was reached. Despite the validation step, the distribution of relevance categories remained unchanged. However, the distribution of data element groups changed. All steps are shown in Figure 1.

### Statistical Analysis

Statistical analysis and graphing were performed using R (R Core Team and the R Foundation for Statistical Computing) [31]. Due to a high number of items in the collection categories such as “Other” or “Other medical history,” we decided to group the corresponding items into new categories. This work was performed by 2 authors to avoid errors.

### Ethical Considerations

Data capture and analysis only included individuals associated with the project. No Institutional Review Board approval was requested, as this would only be appropriate for studies including direct contact with patients [32], but we did not capture or process any patient-related data, so informed consent was not required.

## Results

### Studies Investigated

**Figure 2:**
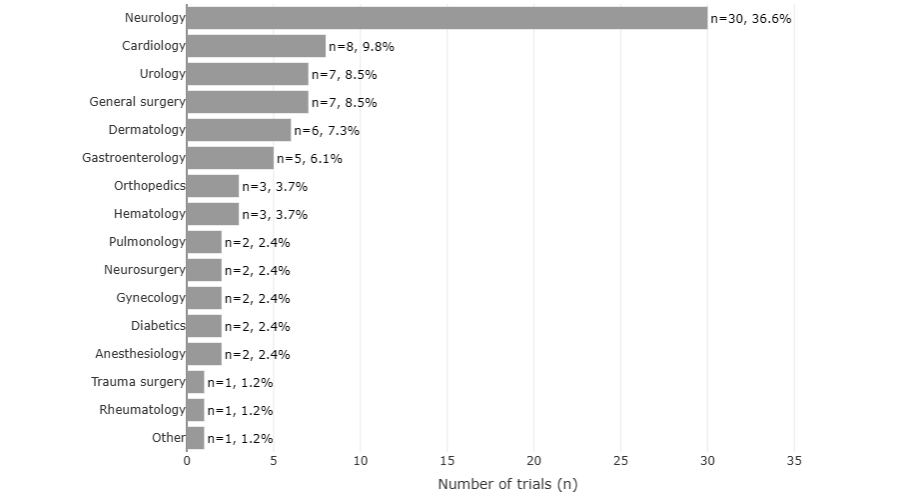
Distribution of the medical specialties among the analyzed studies, sorted in descending order: neurology (n=30, 36.6%), cardiology (n=8, 9.8%), urology (n=7, 8.5%), general surgery (n=7, 8.5%), dermatology (n=6, 7.3%), gastroenterology (n=5, 6.1%), orthopedics (n=3, 3.7%), hematology (n=3, 3.7%), pulmonology (n=2, 2.4%), neurosurgery (n=2, 2.4%), gynecology (n=2, 2.4%), diabetics (n=2, 2.4%), anesthesiology (n=2, 2.4%), trauma surgery (n=1, 1.2%), rheumatology (n=1, 1.2%), and other (n=1, 1.2%).

Figure 2 shows the departments to which the reviewed studies belong. Although no prior decision was made as to which departments to select, there was an overrepresentation of neurological studies.

### Data Element Groups

In total, we included 82 CTs from 8 different university hospitals, and at each site, 3-25 (4%-30%) CTs were processed. In total, we identified 1157 EC, of which 1132 (97.8%) unique criteria remained after the removal of duplicates, which were classified as “irrelevant, redundant.”

**Figure 3:**
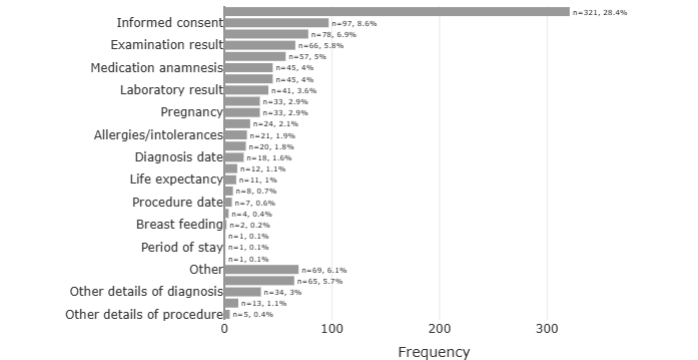
Frequency of data element groups in all EC, in descending order: most frequent were diagnosis (n=321, 28.4%), followed by informed consent (n=97, with 8.6%) and date of birth/age (n=78, 6.9%). EC: eligibility criteria.

Figure 3 shows the frequency of data element groups in all examined EC. With 28.4% (321/1132) of all EC, diagnosis was, by far, the most common data element, followed by informed consent and date of birth/age in second and third places, respectively.

### Relevance Categories

**Figure 4:**
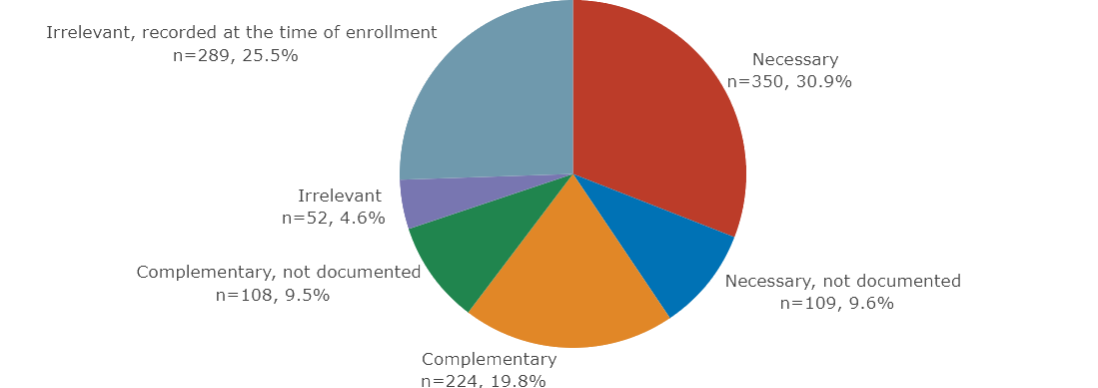
Distribution of relevance categories of all EC: 350 (30.9%) “necessary” criteria, 109 (9.6%) “necessary, not documented,” 224 (19.8%) “complementary,” 108 (9.5%) “complementary, not documented,” 52 (4.6%) “irrelevant,” and 289 (25.5%) “irrelevant, recorded at the time of enrollment.” EC: eligibility criteria.

In terms of EC relevance, 350 (30.9%) of the 1132 EC were categorized as “necessary,” 224 (19.8%) as “complementary,” 217 (19.2%) as “not documented,” 52 (4.6%) as “irrelevant,” and 289 (25.5%) as “irrelevant, recorded at the time of enrollment.” The overall percentages are shown in Figure 4.

#### Total Irrelevant Criteria

In total, 341 (30.1%) EC were categorized as “irrelevant” and “irrelevant, recorded at the time of enrollment.” These were mainly patient consent information (n=90, 26.4%). In addition, the EC “other” (n=42, 12.3%) and “other medical history” (n=26, 7.6%) were often marked as not relevant for patient screening. Diagnosis was only a small part (n=24, 7%) of all irrelevant EC, which is low compared to the general data element distribution shown in Figure 3, where this category makes up the largest part. A visualization of the complete list and frequencies can be found in Multimedia Appendix 2.

#### Nondocumented Criteria

In total, 217 (19.2%) necessary and complementary EC were categorized as not documented and were most commonly the data element groups diagnosis (n=121, 55.8%), examination results (n=36, 16.6%), and procedures (n=28, 12.9%). As noted in the example in Table 2, it is possible that data element groups such as diagnosis were sometimes categorized as documented and sometimes with other categories, which is valid because it may depend on the medical context of what information is documented. All data element groups and their frequencies can be found in Multimedia Appendix 3.

#### All Relevant Criteria

As defined before, both complementary and necessary EC were relevant for an automated search of potential participants, which were 574 in number (Multimedia Appendix 4). The most frequently used data element groups were diagnosis code, which accounted for 44.4% (n=255) of all necessary EC, followed by date of birth (n=74, 12.9%) and procedure codes (n=48, 8.4%).

### Relevance Distribution by Data Element Group

**Figure 5:**
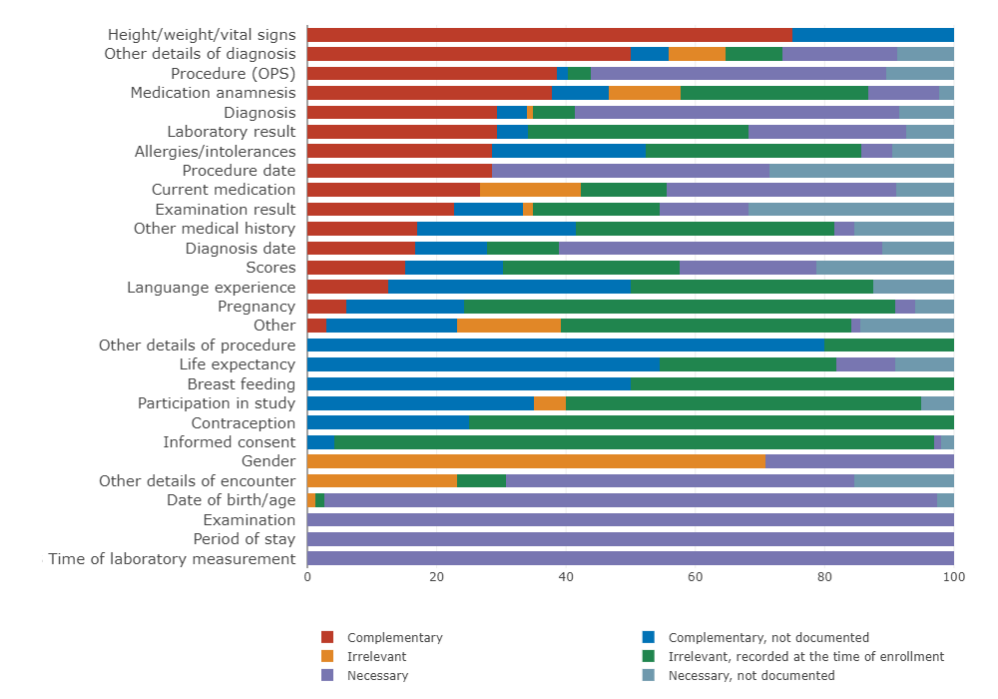
Distribution of relevance categories in percentage by data element group; the groups were recorded at least 5 times in our data set, ordered by the proportion of necessary EC: date of birth/age, other details of encounter, diagnosis, and diagnosis date showed the highest proportion of necessary EC. EC: eligibility criteria.

Since each criterion was individually classified into a data element group and a relevance category, it is possible that common data element groups play a role in several of the categories. For this reason, the categorization between data element groups was heterogeneous. For example, the data element group diagnosis was mostly categorized as necessary or complementary (n=255, 79.4%), while the data element group informed consent was mostly categorized as “irrelevant, recorded at the time of enrollment” (n=90, 92.8%). In addition, the data element groups pregnancy, contraception, and lactation were mostly either not documented or documented at the time of enrollment. Figure 5 shows the relevance distribution for all data element groups.

### Data Element Relevance Index

More important than the absolute frequency of a data element is the question of how many CTs the element is used for in patient screening and not how often a group is represented in a CT. This parameter, the data element relevance index (DERI), can be calculated without considering the frequency of data element groups in CTs.

**Figure 6:**
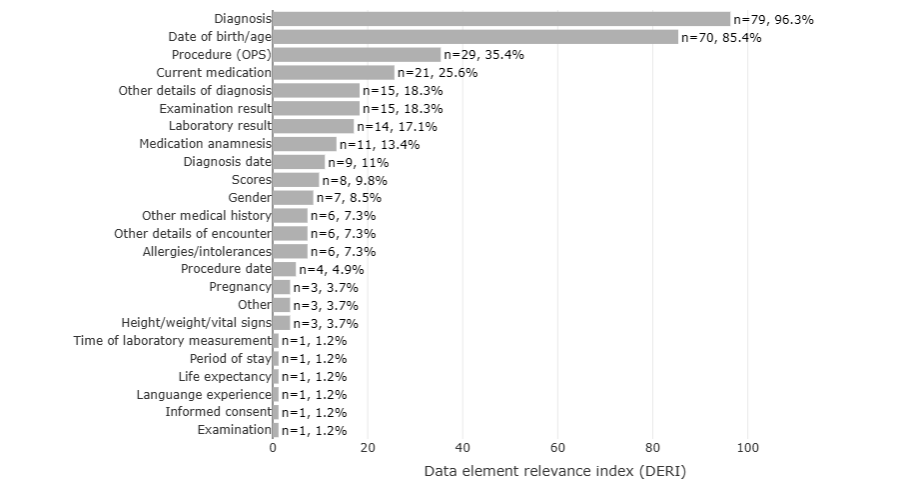
DERI as a percentage of CTs with relevant (necessary or complementary) data element groups by data element group: diagnosis, date of birth/age, and procedure (OPS) show the highest DERI. CT: clinical trial; OPS: *Operationen- und Prozedurenschlüssel*.

For the calculation, we determined the number of CTs in which the data element was used at least once and removed all entries marked as undocumented or irrelevant. The results showed that the data element groups diagnosis and date of birth/age were present in more than 50% of the CTs: diagnosis, n=79 (96.3%); date of birth/age (85.4%). All other DERI values are shown in Figure 6.

### Surrogate Parameters

For the case of unavailability of an original data element, 92 (8.1%) of the EC were documented surrogate parameters to use. Surrogate parameters were often used for the grouping categories “Other medical history” (n=14, 15.2%) and “Other diagnosis information” (n=13, 14.1%). Diagnosis had the highest frequency in both original elements (n=20, 20.7%) and surrogates (n=52, 56.5%). As a surrogate data element for diagnosis, procedures (n=5, 25%) and laboratory results (n=5, 25%) were most often used.

## Discussion

### Principal Findings

EC have been analyzed and categorized repeatedly in recent years to measure their prevalence in CTs. These studies have mostly categorized EC into semantic categories [33]. Comparing the studies, we saw that the frequency of semantic categories shows some overlap but also varies to some extent [28]. This could be due to the selection process of the studies or a different way of categorization by the diverse researchers.

### Data Element Groups

Although the categorization by Luo et al [33] focuses on the semantic categories of EC rather than individual data elements, as in this paper, parallel categories exist that we used to find similarities and differences. Previous studies have measured the prevalence of data element groups as a percentage of all EC examined [25,28-30,33].

Our measured prevalence of similar data element groups differed by no more than 0.5 from the minimum and maximum of the measured frequency percentages of comparable studies. This comparison using the semantic categories described by Luo et al [33] is provided in Multimedia Appendix 5. An exception is the consent category, which we used more often. In our review, consent was often used more than once for a CT, not only to describe consent to the CT itself, but also when a participant was asked to consent to specific procedures or circumstances of the CT. This included, for example, consent to use adequate contraception for the duration of the CT. The high deviation from other studies may be due to the fact that other studies have only used this data category for specific CT consent [25,28,29].

### Relevance Categories

The results show that about 70% of the examined EC are relevant for the selection of potential CT participants. About 19% are not usually documented in EHRs and therefore cannot be used for filtering, even if the trial professional searches all patient records. Whether a criterion is classified as undocumented depends, in part, on the capabilities of hospital information systems and therefore varies from hospital to hospital. In addition, certain information may only be collected depending on the context of care and the severity of illness.

About 51% of all EC are relevant for the electronic prefiltering of patients. Since the implementation of EC is one of the obstacles in the development of a CTRSS, the realization that only about half of the EC are relevant can mean a simplification in the implementation of EC in the systems.

Figure 5 shows that it is not possible to link data element groups only to 1 relevance category. Instead, depending on the context of the CT, different relevance categories were assigned to the data element groups. In some cases, the same group was sometimes categorized as documented and sometimes as undocumented. This can be explained by the fact that whether information is documented depends not only on the data element group but also on several other factors. For example, often, only diagnoses that are considered important and investigated in the context of therapy are documented. In these cases, the trial professionals may be able to make an assessment because they are either part of the patient care or are in close contact with the treatment physicians and are therefore aware of these details for documentation purposes. Figure 5 can provide some guidance as to which data element groups are typically not needed, but an assessment should always be made directly by the trial professionals.

In previously published studies on the prevalence of EC, this was usually also referred to as their relevance. Our results show that a significant proportion of EC is not used to search for participants. Since the classification according to relevance categories varies between the data element groups, as shown in Figure 5, it is apparent that filtering according to relevance criteria affects not only the number of EC but also the frequency distribution of the data element groups.

For this reason, we compared the general frequency of data element groups (Figure 3) with those categorized as necessary or complementary (Multimedia Appendix 4). From this comparison, we saw that the percentage prevalence of all data element groups is sometimes different from the prevalence of the relevant data element groups. Diagnosis and date of birth/age as well as procedures show a particularly strong increase in frequency. However, consent, other medical history, and pregnancy are significantly less frequent among the data elements classified as relevant.

### Data Element Relevance Index

The frequency order of data element groups depends on how they are viewed. If the EC criteria are filtered based on their relevance in prefiltering possible participants, the frequency order changes.

The introduction of a new DERI measurement value changes the perspective. The occurrence frequency of data element groups, such as various laboratory values, in a study is no longer relevant. The consideration now lies only in the frequency of the respective group’s use in studies. Studies with a significant number of exceptional cases exert less influence on the general outcome.

Furthermore, by using the DERI value, it is possible to determine the number of studies reliant on a specific data element group. Consequently, a direct inference can be made about its impact within a CTRSS, which is unachievable through pure frequency data.

### Data Completeness

In 2018, Vass et al [34] examined the data quality of data element groups commonly used in CT EC in 10 university hospitals in Germany. They found that the data completeness is partly heterogeneous and that elements are rarely collected in a structured way in daily clinical practice, which hinders automated retrieval [34].

**Figure 7:**
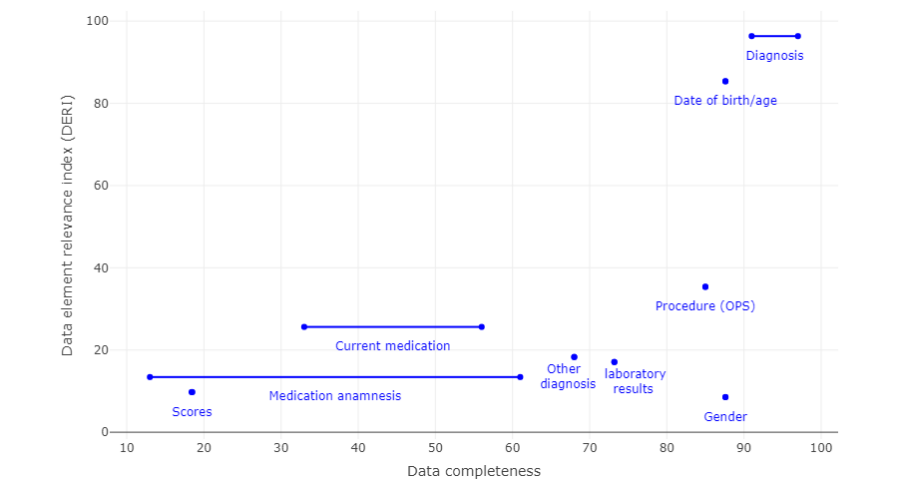
Data completeness measured by Vass et al [34] in comparison with DERI: It can be seen that although diagnosis and date of birth/age both have a high data completeness rate and DERI, problems arise with scores, medication anamnesis, and current medication, which have a low data completeness rate. OPS: *Operationen- und Prozedurenschlüssel*.

Comparing the DERI values of the data element groups and the completeness of data in the EHRs (Figure 7), we saw that more than 80% of the information is available for the 3 most relevant data element groups (diagnoses, demographics, and procedures). The less relevant categories of laboratory results and other diagnostic information are still available in more than 65% of cases. However, medication information (medication history and current medication) is problematic, with availability ranging from 13% to 61%. Nevertheless, medication history and current medication are relevant groups, with DERI values of 13% and 26%, respectively. Scores are also poorly documented, with a data completeness of 18%.

To reliably use these data in a CTRSS, a higher level of completeness should be present for DERI values higher than 10%. Poor data quality of data from EHRs, when matched with EC from CTs, can lead to high false-negative rates for inclusion criteria and high false-positive rates for exclusion criteria. The former, in particular, is fatal to the use of a CTRSS, as matching individuals are thus overlooked. High false-negative rates, in turn, lead to increased workload for trial personnel.

The study by Vass et al [34] showed that some data element groups are well captured in EHRs but other elements are problematic. This generates a data gap between EC and EHRs, which was analyzed by Butler et al [35]. They found that about 40% of all EC are not captured in EHRs. Since a structured collection of clinical patient-related information is important not only for the implementation of a CTRSS but also for billing purposes and patient safety, hospitals as well as governments are pushing the digitalization of patient records. In recent years, many initiatives and laws have been implemented to make data from EHRs available for research purposes [36,37]. Therefore, it is likely that the accessibility of medical information has recently improved or will improve in the future.

The timeliness of data element accessibility was not considered here but should be in future studies to assess not only whether a data element is documented in EHRs but also whether this data element is accessible in time. We should also examine whether the results of Butler et al [35] are reproducible when only relevant criteria are examined.

### Implementation of Patient Recruitment Systems

In previous publications, the relevance of data element groups has been described as the frequency of a group relative to the number of all data elements found in all EC, which depends only on the distribution of data element groups. Since the prevalence of EC is defined heterogeneously in the literature, it is not useful to assess the relevance of data element groups based on the frequency distribution alone.

Especially for the implementation of a CTRSS, it is useful to evaluate how many CTs use a data element group. Using the DERI to determine the relevance of data element groups can be helpful here, as it additionally includes only groups that are used to search for participants.

The core functionality of a CTRSS is to compare EC with clinical data to identify potential participants. For the system to be useful to trial staff, the suggestions must be as accurate as possible, with minimal false-positive or false-negative rates. Since the comparison between the clinical database and EC is the critical factor at this point, it is particularly important that as many EC as possible be checked automatically. When implementing a CTRSS for different types of CTs, it is recommended to first identify important data element groups by determining a local DERI for all data element groups. Therefore, a set of CTs should be analyzed in cooperation with the trial sites, and all data element groups with a high DERI should be accessible to the CTRSS. For this purpose, it is useful to set a threshold value for the DERI determined and to make available all data element groups that exceed this value. For this purpose, we could start with a value of 10, since this means that a data element group is used in at least 10% of the CTs, and gradually increase this value. Further studies are needed to determine the quantitative relationship of the DERI to the results of a CTRSS.

In contrast to the determination of local DERI values, there is the additional task of determining the data quality of the available data sources to be used for matching with the EC of CTs. Again, data completeness and timeliness should be determined locally to ensure that the necessary data element groups identified are available in the highest-possible data quality.

### Limitations

Since the PPs at the study sites were allowed to select participants themselves, the selection of participants was through the convenience sampling method. Randomization was not possible; instead, all participants who were currently supervising at least 1 CT and who were willing to participate in the study were included. Additionally, we could see an overrepresentation of neurological CTs in our sample and had to exclude oncological and psychiatric CTs.

The categorization of EC was performed with the cooperation of CTRSS experts and trial professionals at each site. To minimize the bias of different categorization methods, all the categorization of data element groups was validated by 2 experts.

The results presented here are dependent on the study sites selected. The clusters we identified may be prone to local variation, and their applicability to other sites is unclear.

### Conclusion

In this study, we demonstrated that automated recruitment support of CT personnel requires only roughly 50% of the EC indicated in the CT protocols. Since the frequency of EC in CTs is described differently in the literature, exclusively focusing on the frequency of EC is misleading. Instead, we propose to define the relevance of EC as the proportion of CTs in which a criterion occurs. In addition, only EC considered relevant (necessary or complementary) for patient recruitment should be included for this determination. This DERI can be used to quantify the relevance of EC data element groups.

Further examination is necessary to find out whether the relevant data element groups are documented in EHRs in a structured way and accessible in time for the implementation of a CTRSS.

## Appendix

Multimedia Appendix 1 Captured information.

Multimedia Appendix 2 Irrelevant data element groups with frequency.

Multimedia Appendix 3 Not-documented data element groups with frequency.

Multimedia Appendix 4 Relevant data element groups with frequency.

Multimedia Appendix 5 Comparison of data element groups' distribution with other studies.

Multimedia Appendix 6 All analyzed eligibility criteria of all included trials.

## Abbreviations

CT: clinical trial
DERI: data element relevance index
EC: eligibility criteria
EHR: electronic health record
OPS: *Operationen- und Prozedurenschlüssel*
PP: project participant

## Data Availability

We added the parameters of our data collection sheet, as well as all analysis and results, either in this paper or in the appendices. Additionally, all data our analysis is based on can be found in Multimedia Appendix 6. We only had to remove original trial descriptions, as they would allow a conclusion on concrete clinical trials and locations.
