# Supplementary material for: Evaluation of Eligibility Criteria Relevance for the Purpose of IT-Supported Trial Recruitment: Descriptive Quantitative Analysis
Source: JMIR Form Res. 2024 Jan 31;8:e49347. doi: 10.2196/49347 (PMC10867759; doi:10.2196/49347)
Supplement: Multimedia Appendix 1 [file formative_v8i1e49347_app1.docx]

# Appendix 1: Captured Information from Data Collection Sheet

| **Original description** | free text |
| --- | --- |
| **Content (simplified)** | free text |
| **Data Element** | Selection from data element list:   - allergies/intolerances - laboratory examination - current medication - date of birth/age - diagnosis - diagnosis date - examination - examination result - gender - height/weight/vital signs - informed consent - laboratory result - medication anamnesis - other - other medical history - other details of diagnosis - other details of encounter - other details of procedure - participation in study - period of stay - pregnancy - procedure (OPS) - procedure date - scores - time of laboratory measurement |
| **Relevance assessment** | Selection from:   - necessary - necessary - not documented - complementary - complementary - not documented - irrelevant - irrelevant - recorded at time of enrolment - irrelevant - redundant |
| **Surrogate parameter** | Selection from data element list (s.a.) |
| **Comment** | free text |
