# Supplementary figures and images for: Evaluation of Eligibility Criteria Relevance for the Purpose of IT-Supported Trial Recruitment: Descriptive Quantitative Analysis
Source: JMIR Form Res. 2024 Jan 31;8:e49347. doi: 10.2196/49347 (PMC10867759; doi:10.2196/49347)

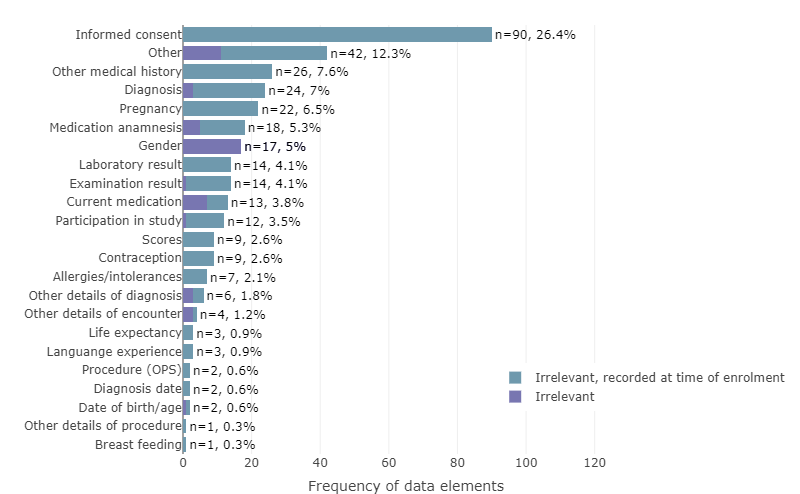

Supplement: Multimedia Appendix 2 [file formative_v8i1e49347_app2.png]

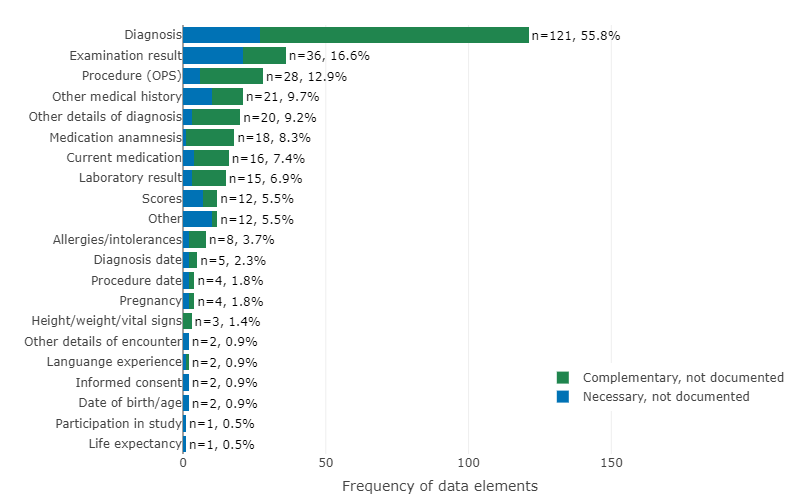

Supplement: Multimedia Appendix 3 [file formative_v8i1e49347_app3.png]

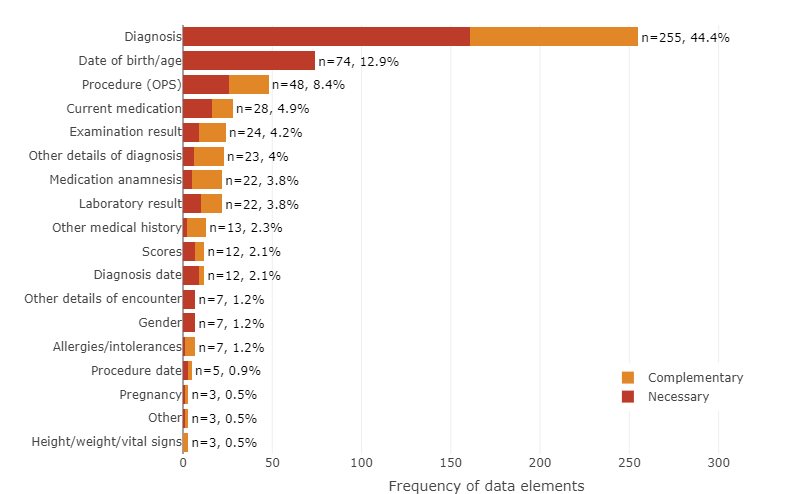

Supplement: Multimedia Appendix 4 [file formative_v8i1e49347_app4.png]

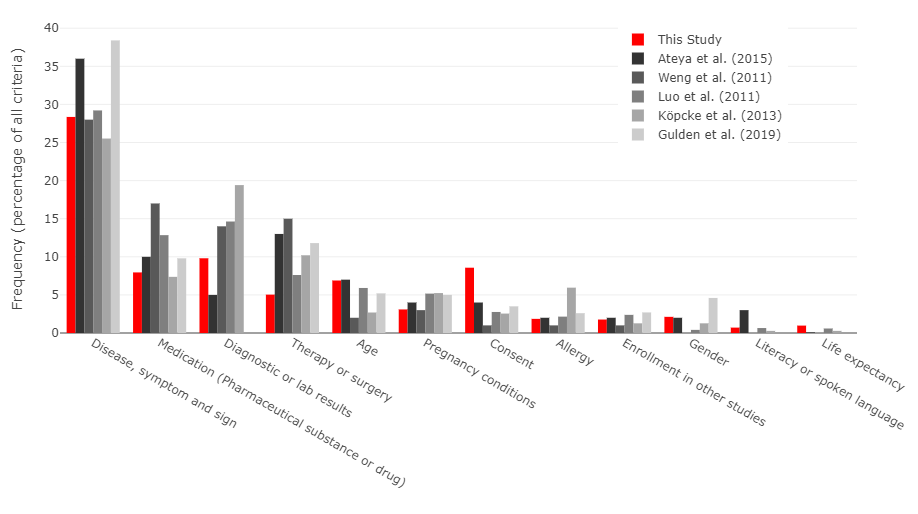

Supplement: Multimedia Appendix 5 [file formative_v8i1e49347_app5.png]
